# Recent Insights Into the Multiple Pathways Driving Non-alcoholic Steatohepatitis-Derived Hepatocellular Carcinoma

**DOI:** 10.3389/fonc.2019.00762

**Published:** 2019-08-13

**Authors:** Kazuki Takakura, Tsunekazu Oikawa, Masanori Nakano, Chisato Saeki, Yuichi Torisu, Mikio Kajihara, Masayuki Saruta

**Affiliations:** Division of Gastroenterology and Hepatology, Department of Internal Medicine, The Jikei University School of Medicine, Tokyo, Japan

**Keywords:** hepatocellular carcinoma, non-alcoholic steatohepatitis, non-alcoholic fatty liver disease, tumor necrosis factor, dysbiosis, signal transducer and activator of transcription

## Abstract

The incidence of metabolic syndrome with fatty liver is spreading on a worldwide scale. Correspondingly, the number of patients with the hepatic phenotype of metabolic syndrome, non-alcoholic fatty liver disease (NAFLD), and in its advanced states, non-alcoholic steatohepatitis (NASH), and the subsequent hepatocellular carcinoma (HCC) derived from NASH (NASH-HCC) is increasing remarkably. A large-scale epidemiological study revealed that obesity can be a risk factor of such cancers as HCC. Moreover, despite the ongoing trends of declining cancer incidence and mortality for most cancer types, HCC has experienced a markedly increased rate of both. Considering the differences in liver-related mortality among NAFLD patients, NASH, and NASH-HCC should be included in the objectives of initiatives to manage NAFLD patients and their progression to the advanced stages. Unfortunately, research has yet to make a crucial drug discovery for the effective treatment of NASH and NASH-HCC, although it is urgently needed. The latest widespread concept of the “multiple parallel hits hypothesis,” whereby multiple factors contribute concurrently to disease pathogenesis has led to advances in the elucidation of hepatic and systemic molecular mechanisms driving NASH and the subsequent NASH-HCC progression; the results are not only extensive but promising for therapeutics. Here, we have summarized the myriad landmark discoveries of recent research into the pathogenic processes underlying NASH-HCC development and with the greatest possibility for a new generation of pharmaceutical products for interference and treatment.

## Introduction

Compared with viral or alcoholic chronic hepatitis, non-alcoholic fatty liver disease (NAFLD) is becoming a major etiology of hepatocellular carcinoma (HCC), based on the rising prevalence of obesity on a global scale, and especially in developed countries (1), which account for at least 30% of the estimated ratio of adult population with NAFLD (2). A large-scale epidemiological study revealed that obesity can be a risk factor of such cancers as HCC (3). Moreover, despite the ongoing trends of declining cancer incidence and mortality for most cancer types, HCC has experienced a markedly increased rate of both (4). As the incidence of metabolic syndrome related to obesity is widely spreading, so is the number of patients with NAFLD and its advanced states, non-alcoholic steatohepatitis (NASH), and its subsequent NASH derived hepatocellular carcinoma (NASH-HCC). Therefore, an effective treatment for NASH and NASH-HCC is urgently required, and researchers continue their efforts toward the crucial drug discovery.

Reflecting the classical concept of the “two-hit hypothesis” which has been insufficient to illustrate the various molecular and metabolic involvements in NAFLD/NASH-HCC progression (5), the “multiple parallel hits hypothesis” has become recognized as underlying the pathophysiology of NASH and its progression to NASH-HCC (6). Based on this recently-applied concept, the current research findings have advanced our knowledge of the hepatic and systemic molecular mechanisms driving NASH and NASH-HCC progression, and the results have not only been extensive but are also promising for therapeutics. Indeed, there have been several studies which have provided reliable scientific validation to the idea of progression from fatty liver to NASH-HCC.

Oxidative stress and lipotoxicity have been demonstrated to play an important role in the progression of NASH and fibrosis (7). The oxidative stress promotes hepatocyte cell death and activation of inflammatory pathways, including expression of the pro-inflammatory cytokine tumor necrosis factor (TNF), that lead to advanced fibrosis and cirrhosis (7, 8). The relationship between a disturbance of gut microbiota and NASH-HCC development was recently elucidated. Moreover, another recent study identified the independent regulation of NASH and NASH-HCC by the activated signal transducer and activator of transcription (STAT) signaling pathways (9).

These current advances in our understanding with several helpful review papers regarding the pathogenic mechanisms underlying the NASH-HCC developmental process (10–12), along with the findings that are forthcoming from ongoing clinical trials, will open new avenues toward the discovery of the next generation of pharmaceutical products for treating this deadly disease.

## Inflammatory Mediators Centered Upon TNF

Substantial aspects of NASH progression are the precursor aggregation of immune cells (i.e., Kupffer cells, macrophages, B cells, T cells, etc) and the successive induction of hepatic inflammation, triggering the recurrent cycle of tissue damage and repair (7, 8). A study using a NASH mouse model showed that the TNF-α signal derived from Kupffer cells in the liver plays a pivotal role in NASH development (13). Furthermore, another murine model-based study clearly demonstrated that the TNF derived from inflammatory liver macrophages is indispensable for NASH and steatohepatitic HCC development under high-fat diet (HFD) feeding conditions in MUP-urokinase plasminogen activator (commonly known as uPA) mice (14); this effect occurs through the transient endoplasmic reticulum (ER) stress response, which enhances lipogenesis and increases the degree of hepatic steatosis (15–17).

Several potential mechanisms for the triggering of HCC development *via* the comorbid condition of ER stress and HFD feeding have been proposed. First, HFD can sustain a modest degree of ER stress in MUP-uPA mice by stimulating hepatosteatosis. Second, ER stress can promote the enhancement of lipogenesis and hepatic steatosis. Third, ER stress and steatosis can increase production of reactive oxygen species (ROS) in hepatocytes, causing oxidative stress and subsequent genomic instability. Fourth, ER and oxidative stress can stimulate the sensitivity of hepatocytes to lipotoxic death, thereby releasing inflammatory mediators. Fifth, TNF and other mediators produced by activated inflammatory macrophages can stimulate compensatory hepatocyte proliferation and expand HCC progenitors; TNF is already known to further reinforce the inflammatory microenvironment and induce expression of related chemokines and growth factors/cytokines.

**Figure 1:**
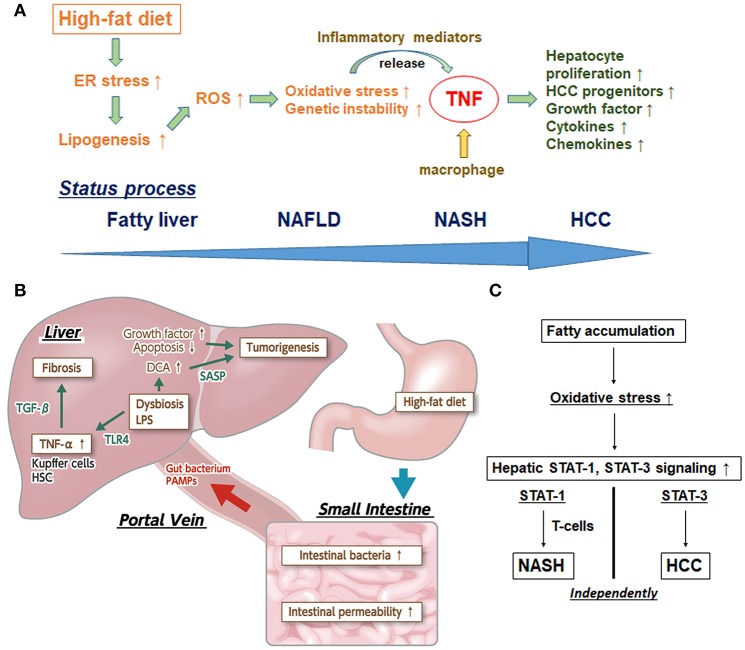
**(A)** Inflammatory pathway centered upon TNF in the process of NASH-HCC development. First, high-fat diet sustains endoplasmic reticulum (ER) stress. Second, ER stress promotes the enhancement of lipogenesis and hepatic steatosis. Third, ER stress and steatosis increase reactive oxygen species (ROS) production to cause oxidative stress and the subsequent genomic instability. Fourth, ER and oxidative stress stimulate release of inflammatory mediators. Fifth, tumor necrosis factor (TNF) produced by macrophages stimulate hepatocyte proliferation and expand HCC progenitors. TNF further reinforces the inflammatory microenvironment and induces expression of related chemokines and growth factors/cytokines. **(B)** The effects of dysbiosis on liver fibrosis and carcinogenesis through the microbiota-liver axis. Several gut-derived factors, including gut bacteria, pathogen-associated molecular patterns (PAMPs), etc, have a strong impact on hepatic diseases *via* the portal vein. The intestinal permeability is also significantly enhanced, and aberrant overgrowth of intestinal bacteria has been found in most patients with NAFLD and NASH. In addition, lipopolysaccharide (LPS) in portal blood supports the augmentation of TNF-α production in Kupffer cells, through an enhanced toll-like receptor 4 (TLR4) signal. LPS-enhanced sensitivity to transforming growth factor-beta (TGF-β) results in progression toward liver fibrosis and promotion of tumorigenesis in the liver. Augmented deoxycholic acid (DCA) has been shown to cause hepatic stellate cells' senescence, thereby facilitating hepatocarcinogenesis *via* the senescence-associated secretory phenotype (SASP) factor. **(C)** Individual pathways of STAT-1-dependent NASH and STAT-3-dependent HCC from fatty liver. Activation of protumorigenic signaling pathways is stimulated in HCC. Signal transducer and activator of transcription-3 (STAT-3) signaling is key in driving the transformation of tumor progenitors and progression to HCC. The dissociation between NASH and HCC in obesity depends upon different STAT signaling pathways. Stimulated STAT-1 leads to cytotoxic T cell activation in NASH but not in HCC; in contrast, STAT-3 is responsible for T cell protein tyrosine phosphatase (TCPTP) inactivation that promotes HCC in obesity, independent of T cell recruitment.

Importantly, it is the interplay of these factors that facilitates the developmental process to NASH-HCC. Complementarity of ER stress and hepatosteatosis are needed for the pathogenesis process (18). Altogether, however, TNF plays a mandatory role with affecting various related molecules in the inflammatory pathways underlying NASH-HCC progression, and anti-TNF drugs may be promising for fighting NASH and steatohepatitic HCC progression (Figure 1A).

## Dysbiosis Through the Microbiota-Liver Axis

### Enterohepatic Correlation

Due to the anatomical position of the liver, several gut-derived factors, including gut bacteria, and pathogen-associated molecular patterns (known as PAMPs), exert a strong impact on hepatic diseases *via* the portal vein. Therefore, dysbiosis (the collective term for the collapse of gut homeostasis) is expected to have an intimate involvement in NASH progression. Indeed, patients with NAFLD show significantly increased endotoxin concentration in peripheral blood and significantly enhanced intestinal permeability (19, 20). Furthermore, it was reported that most of the patients with NAFLD and NASH have an aberrant overgrowth of intestinal bacteria (20–22) as well as detectable lipopolysaccharide (commonly referred to as LPS) in portal blood (23); the latter will serve to augment TNF-α production in Kupffer cells through enhancement of the Toll-like receptor (TLR) 4 signal. In addition, the LPS-stimulated gut-activated TLR4 signal, not only in Kupffer cells but also in hepatic stellate cells, and the enhanced sensitivity to transforming growth factor-β result in progression of liver fibrosis (24). Taken together, the intestinal permeability, aberrant overgrowth of intestinal bacteria, and inflow of PAMPs *via* the gut-liver axis directly modify the NAFLD/NASH status by stimulating the hepatic innate immune system.

### Metagenome Analyses in NAFLD and NASH

Recently, a fascinating study of obese children showed that intestinal bacterial groups of Proteobacteria, Enterobacteria, and Escherichia were significantly increased in those children with NASH, as compared to those without (25). Another comparative study found that intestinal bacterial groups of Firmicutes, Lachnospiraceae, Lactobacillaceae, and Lactobacillus were markedly augmented in obese patients with NAFLD compared to healthy controls (26). However, the data of these metagenome analyses can be conflicted due to the small number of included patients and their differences in race, sex, age, and geographic region. Thus, a large-scale study controlled for background factors is required.

Additionally, activation of dysbiosis through the nucleotide-binding domain-like receptor protein (NLRP) 3 inflammasome or NLRP6 inflammasome and interleukin (IL)-18 production was elucidated (27, 28). Moreover, a study of NLRP3- and NLRP6-deficient NASH model mice showed that promoting dysbiosis initiated colitis through the secretory C-C motif chemokine ligand 5 (commonly known as CCL5); ultimately, the consequent increased amounts of TLR4 ligand and TLR6 ligand reaching the liver *via* portal blood and the enhanced hepatic TNF-α production resulted in acceleration of the NASH state (27, 28).

Further, it was reported that HFD caused decrease of Bacteroidetes and increase of Proteobacteria and Firmicutes in obese mice independent of weight gain and based upon the great impact of diet on dysbiosis (29). Yet another murine-based study showed that dysbiosis worsens hepatic fibrogenesis (30). In sum, these metagenome analyses of NAFLD and NASH have affirmed the bench science investigations of the relation to dysbiosis.

### Tumorigenesis With Gut Bacterium

A previous study demonstrated that LPS, a TLR4 ligand derived from intestinal flora, promoted tumorigenesis in liver by facilitating the production of downstream growth factors, including epiregulin, and by inhibiting apoptosis in the setting of significant inflammation (31).

Otani et al. (32) found that augmenting deoxycholic acid caused hepatic stellate cells to enter senescence, thereby facilitating hepatocarcinogenesis by senescence-associated secretory phenotype factor (known as SASP) secreted from the hepatic stellate cells, upon the consequent promotion of deoxycholic acid (a secondary bile acid) production *via* increasing intestinal flora classified in the Clostridium cluster XI bacterial family. Because all of the above studies confirmed the marked inhibition of tumor formation in liver upon intestinal sterilization that occurs with administration of antibiotics, it is probable that gut microbiota is involved in NASH-HCC progression (Figure 1B) and can be a potential therapeutic target for steatohepatitic HCC.

### Tumor-Promoting Signaling Pathways Focused on STAT

An unanswered question related to hepatocarcinogenesis relates to the crucial difference in the liver background, being with or without cirrhosis or advanced fibrosis. Indeed, there are several reports which have shown the development of HCC from NAFLD in patients without cirrhosis or fibrosis (1, 33–38), whereas HCC typically occurs in the cirrhotic or fibrotic condition (7). The activation of pro-tumorigenic signaling pathways, such as IL-6 and Janus-activated kinase (known as JAK)-STAT signaling pathways, were determined to be stimulated in HCC by over 28,000 mutations (39).

Intriguingly, STAT-3 signaling was found to be key in driving the transformation of tumor progenitors and HCC progression in animal models (40–43). Further, it has been reported that STAT-3 is activated in most human HCCs and presents positive correlation with tumor malignancy (40, 44). Grohmann et al. (9) recently reported a groundbreaking analysis of the dissociation between NASH and HCC in obesity depending on different STAT signaling pathways. In brief, stimulated STAT-1 lead to cytotoxic T cell activation in NASH but not in HCC, while STAT-3 was responsible for T cell protein tyrosine phosphatase (known as TCPTP) inactivation promoting HCC in obesity, independent of T cell recruitment (Figure 1C). This mechanistic breakthrough in the understanding of NASH-HCC progression can serve as evidence for the incidence of HCC in NAFLD without cirrhosis.

### Genetic Involvement

There is a virtual certainty that genetics have an intimate involvement in the pathogenesis of NAFLD. Genome-wide association studies have shown that single nucleotide polymorphisms in the patatin-like phospholipase domain-containing protein 3 gene (rs738409 encoding I148M and rs6006460 encoding S453I alleles of PNPLA3) are related to NAFLD (45) and NASH (46), suggesting a relationship between PNPLA3 and hepatocarcinogenesis. Actually, a Japanese group subsequently demonstrated the rs738409 variant as a major risk factor for development of HCC in patients with type 2 diabetes mellitus, a condition closely associated with NASH (47).

In addition, a recent study demonstrated that cytosine DNA methylation which acts as a transcriptional “ON-OFF” switch and a specific over-expression of tubulin beta 2B class IIB (*Tubb2b*) mediated by aberrant DNA methylation are closely associated in the development of NASH-HCC in a mouse model (48). It indicates that the involvement of epigenetic mechanisms in the regulation of *Tubb2b* expression in the pathogenesis of NASH-HCC.

### Autophagy

Macroautophagy [autophagy; a major cellular degradative system which eliminates unneeded or dysfunctional organelles (49)] can also influence NAFLD, NASH and NASH-HCC (50–55). Mathew et al. (56) demonstrated that metabolic stress caused autophagy-defective tumor cells to accumulate p62 (an autophagy substrate), resulting in promotion of retainment of damaged mitochondria, elevated oxidative stress, and activation of the DNA damage response, suggesting that autophagy suppresses tumorigenesis through elimination of p62. Moreover, a Japanese group showed that Rubicon (a suppressor of the late stage of autophagy) is elevated in NAFLD and is responsible for accelerating hepatocellular fat accumulation and apoptosis (57). These collective findings suggest that NASH-HCC development are associated with an autophagy defect and genetic and/or autophagic modulation may be novel therapeutic targets for managing NASH and NASH-HCC development.

### Clinical Trials and Future Direction

NASH development appears to be dependent on multiple factors working in parallel (i.e., the “multiple parallel hits hypothesis”). These factors include microbiota-related factors, HFD additives, dysbiosis, IL-6 and TNF from adipose tissue, and mitochondrial dysfunction and oxidative or ER stress in the absence of identified genetic factors (6, 58). Moreover, NASH and HCC progressions proceed separately *via* different STAT signaling pathways, as recently demonstrated. The data from that groundbreaking study provided an answer to a question on how HCC develops under disparate liver backgrounds (i.e., with or without cirrhosis or fibrosis). In particular, it suggests that interaction with multiple pathways' blockade or inhibition should be theoretically needed as the base for the next generation of pharmaceutical products addressing NASH and NASH-HCC individually. In addition, it is presumed that steatohepatitic HCC with cirrhosis or severe fibrosis can be well-managed along with NASH itself through targeting of the STAT-1 signaling pathway, whereas NASH-HCC without cirrhosis or fibrosis would be treated independently through the STAT-3 signaling pathway. Giving comprehensive consideration to the overall survival of NASH patients, the potential therapeutic effects obtained through the STAT-1 or STAT-3 signaling pathway can also serve to prevent advancement to liver failure as well as hepatocarcinogenesis. Indeed, a phase I/Ib Study of AZD9150, antisense oligonucleotide inhibitor of STAT-3, in patients with advanced/metastatic HCC (NCT01839604) and a phase I study of OPB-111077, an oral STAT-3 inhibitor, in subjects with advanced HCC (NCT01942083) were certainly conducted, although the efficacy outcomes were limited (59).

Since NASH is regarded as a part of systemic metabolic abnormality, continuous lifestyle modification with dietary caloric restriction and exercise is fundamentally important to its therapeutic management, albeit an arduous task for many patients. Based on the interpretation of the therapeutic effect for underlying disease exerting beneficial effects on NASH, several clinical trials are ongoing with NAFLD/NASH patients on various treatment regimens targeting the former, including anti-diabetic drugs, anti-hypertensive drugs, and lipid-lowering drugs (Table 1). Other clinical trials for NASH patients using antibiotics and anti-fibrotic agents are also actively ongoing. Further investigation in the anti-tumorigenic effect of these drugs will be a potential avenue for new drug discovery for steatohepatitic HCC. Reflecting the limited therapeutic effects of various single agents which have been tested in previous and ongoing studies related to NASH and NASH-HCC, with most recent clinical trial in failure of selonsertib (SEL; GS-4997), apoptosis signal-regulating kinase 1 (ASK1) inhibitor, it might be assumed that the analysis for the synergy effects of multidrug administration against NASH and NASH-HCC will be planned in the near future. As genetic and epigenetic factors, gut microbiota, oxidative stress, and autophagy, etc. are variously involved in the pathogenesis, the inhibitory effect to a single factor is limited. Therefore, simultaneous administration of several agents, for example anti-fibrotic drug with anti-diabetic drug, or antibiotic drug with anti-inflammatory drug for NASH-HCC patients would have a high possibility and be of particular interest. Specifically, the distinct amelioration from the advanced NASH state and/or the significant anti-tumor effect against NASH-HCC by some combined therapies without producing any serious adverse events are exactly what we expect. Thus, if the synergistic effects of the simultaneous multiple pathways' blockade is confirmed and if how the different pathways are differentially regulated in HCC vs. NASH-induced HCC would be more clearly, the more effective combination usage can be a candidate approach for the next generation of pharmaceutical products for NASH-HCC.

**Table 1:** Current clinical trails for NAFLD/NASH patients.

**Trial identifier**	**Target**	**Targeted agent**	**Type of targeted drug**	**Category of agent**	**Enrollment**	**Organizing location**	**Study phase**
NCT02970942	NASH	Semaglutide	GLP-1 RA	Anti-diabetic drug	288	Globally	Phase 2
NCT02696941	NAFLD	Dapagliflozin, Metformin	SGLT2 inhibitors, Biguanide	Anti-diabetic drug	20	United Kingdom	Phase 1
NCT02875821	NAFLD	Lpragliflozin	SGLT2 inhibitors	Anti-diabetic drug	44	Republic of Korea	Phase 4
NCT02964715	NAFLD	Empagliflozin	SGLT2 inhibitors	Anti-diabetic drug	25	Malaysia	Phase 4
NCT02279524	NASH	Aramchol	Cholic-arachidic acid conjugate	Lipid-lowering drug	247	Globally	Phase 2, 3
NCT02856555	NASH	GS-0976	Acetyl-Goa carboxylase	Lipid-lowering drug	127	United States Australia, Israel	Phase 2
NCT02316717	NASH	IMM-124E	Bovine colostrum	Antibiotic drug	133	United States	Phase 2
NCT02510599	NASH	Solithromycin	macrolide	LPS-induced antibiotic drug	10	United States	Phase 2
NCT02442687	NASH	JKB-121	TLR4 antagonist	Anti-inflammatory drug	65	United States	Phase 2
NCT03028740	NASH	Cenicriviroc	CCR2/5 antagonist	Anti- fibrotic drug	2000	Globally	Phase 3
NCT02462967	NASH	GR-MD-02	Galectin-3 inhibitor	Anti-fibrotic drug	162	United States	Phase 2
NCT02227459	NASH	ND-L02-s0201	Vitamin A-coupled lipid nanoparticle containing siRNA against HSP47	Anti-fibrotic drug	25	United States	Phase 1
NCT03053063	NASH	Selonsertib	ASK1 inhibitor	Anti- fibrotic drug	877	United States	Phase 3

*GLP-1 RA, glucagon-like peptide- 1 receptor agonists; SGLT2, sodium-glucose contransporter 2; TLR4, Toll-like receptor 4; LPS, lipopolysaccharide; CCR2 5, C-C motif chemokine receptor-2/5; ASK1, apoptosis signal-regulating kinase 1*.

## Conclusion

Based on the latest concept of the “multiple parallel hits hypothesis,” the elucidation of hepatic and systemic molecular mechanisms driving patients with NAFLD toward NASH and the subsequent HCC progression are extensive and promising. Several of the advances in our understanding of the systemic molecular mechanisms of NASH-HCC progression provided by the ongoing clinical trials of NASH patients will give clues to the direction of future works, the finding of which would be a long-awaited harvest for the unmet medical needs.

## Author Contributions

KT wrote the manuscript. TO, MN, and CS critically appraised the manuscript. YT, MK, and MS formatted and edited the final manuscript.

### Conflict of Interest Statement

The authors declare that the research was conducted in the absence of any commercial or financial relationships that could be construed as a potential conflict of interest.
